# Selective classification with machine learning uncertainty estimates improves ACS prediction: A retrospective study in the prehospital setting

**DOI:** 10.21203/rs.3.rs-4437265/v1

**Published:** 2024-06-05

**Authors:** Juan Jose Garcia, Rebecca Kitzmiller, Ashok Krishnamurthy, Jessica K. Zégre-Hemsey

**Affiliations:** 1 University of North Carolina at Chapel Hill, Department of Computer Science, Chapel Hill, 27514, United States; 2 University of North Carolina at Chapel Hill, School of nursing, Chapel Hill, 27514, United States; 3 Renaissance Computing Institute, Chapel Hill, 27517, United States

## Abstract

Accurate identification of acute coronary syndrome (ACS) in the prehospital sestting is important for timely treatments that reduce damage to the compromised myocardium. Current machine learning approaches lack sufficient performance to safely rule-in or rule-out ACS. Our goal is to identify a method that bridges this gap. To do so, we retrospectively evaluate two promising approaches, an ensemble of gradient boosted decision trees (GBDT) and selective classification (SC) on consecutive patients transported by ambulance to the ED with chest pain and/or anginal equivalents. On the task of ACS classification with 23 prehospital covariates, we found the fusion of the two (GBDT+SC) improves the best reported sensitivity and specificity by 8% and 23% respectively. Accordingly, GBDT+SC is safer than current machine learning approaches to rule-in and rule-out of ACS in the prehospital setting.

## Introduction

Accurate identification of acute coronary syndrome (ACS) in the prehospital setting is important for timely treatments that reduce damage to the compromised myocardium. Accordingly, the community has developed Machine Learning (ML) methods to improve the prediction of ACS with prehospital covariates^1,2^. Nevertheless, performance remains insufficient for safe rule-out or rule-in of ACS^3^. Current research in cardiovascular disease detection from ECG has observed a possible trade-off between performance and coverage (i.e. percentage of cases to automatically classify) as a viable way to mitigate errors^4–6^. This trade-off is known as selective classification^7,8^ and it provides more accurate predictions by identifying a subpopulation better suited for automatic classification^4,5,9^. In our work, we evaluate a cutoff in the predictive uncertainty of an ensemble of gradient boosted decision trees (GBDT^10^) as the filter for selective classification. We observe an 8% increase in sensitivity and a 23% increase in specificity, at the cost of 25% coverage. More concretely, our contributions are:

1. Identification of a ML model that achieves the best reported ACS prediction performance in the prehospital setting. [See tables 1, 2, 4 and 5]
2. Empirical evidence that selective classification boosts performance at the expense of 25% coverage in the prehospital setting. [See tables 2 and 4]

## Results

### Patient characteristics and outcomes

Data was collected by Zègre-Hemsey and colleagues^11^. Patients enrolled (n=3646) over 21 years old, transported by ambulance to the ED with non-traumatic chest pain and/or anginal equivalents. Emergency healthcare personnel collected clinical information in the ambulance (i.e. Prehospital setting). The primary outcomes recorded any ACS event (i.e. the acute manifestation of coronary heart disease and include ST-elevation myocardial infarction (STEMI), non-ST elevation myocardial infarction (NSTEMI), and unstable angina (UA)). The observed prevalence was ACS (20%), STEMI (14%), NSTE-ACS (7%) and unstable angina (3%). These events are within 30 days post ED admission.

### Dataset derivation and preparation

We divide the dataset into two cohorts: An internal cohort (n=1756 cases before 06/2016) for training and validation, and an external cohort (n=1127 cases after 06/2016) for testing. Furthermore, we select 23 covariates (see table 1) commonly associated with ACS^12^ and available in the prehospital setting^13^. We discarded patients with a missing initial troponin value (25 total) or without an ECG date; less than 2% of patients had missing covariates imputed with a constant^14^.

### Ensemble of Gradient Boosted Decision Trees (GBDT)

(Malinin et al., 2021)^10^ first proposed GBDT to classify tabular data and improve predictive uncertainty estimates. Accordingly, we chose this method because our data is tabular (See table 1) and we use estimates of predictive uncertainty to filter-out patients unsuitable for automatic classification. In this work, the estimate of uncertainty used to make the classification is known as “Total Uncertainty”^15^, and corresponds to the entropy of the posterior predictive distribution *H*(*Y*|*X,D*); where *X* is the test input covariates, *Y* is the unknown outcome (i.e. {*ACS*,¬*ACS*}), *D* is our training split and *H*(·) is the entropy function. Total uncertainty is estimated by a Monte-Carlo approximation^10^:

(1) $$H(Y\left|X,D)=H(E_{\theta |D}[p(Y\right|X,\theta )])$$

(2) $$E_{\theta |D}[p(Y|X,\theta )]\approx \frac{1}{M}\overset{M}{\underset{m-1}{\sum }}p(Y|X,\theta^{\left(m\right)})$$

Note ${\left\{\theta^{\left(m\right)}\right\}}_{m=1}^{M}$ corresponds to an ensemble of *M* Gradient Boosted Trees (GBT) parametrized by *θ*^(*m*)^. Each *θ*^(*m*)^ is sampled i.i.d. from an approximate distribution *q*(*θ*) which converges weakly to a posterior distribution *p*(*θ*|*D*). *p*(*Y*|*X,θ*^(*m*)^) corresponds to the output of a GBT (i.e. a function of *X* that outputs a distribution over a labels *Y*). Note the GBT algorithm is not the standard one, it is modified to guarantee weak convergence. Please see Section 3 from the original paper^10^ for more details. It is worth mentioning that total uncertainty decomposes into two non-negative quantities: Model uncertainty and data uncertainty^15^.


(3) $$H(Y\left|X,D)=I(Y,\theta \right|X,D)+E_{\theta |D}[H(Y|X,\theta )])$$

Intuitively, model uncertainty (i.e. *I*(*Y,θ*|*X,D*)) is high when the input (*x*) is sufficiently different from our training set (*D*) (e.g. Cases with age < 5). On the other hand, data uncertainty is high when the input is inherently random (e.g. Cases with no ST-Elevation). *I*(·,·) represents mutual information.

### Selective classification

Selective classification (SC)^7,8^ filters-out cases at test time with the goal of improving predictive performance over the filtered-in subpopulation. In this work, our filter rule is “Total uncertainty greater than cutoff value” (i.e. *H*(*Y*|*X,D*) > cutoff). We use the validation split (*D*_val_), disjoint from *D*, to determine a total uncertainty cutoff such that 80% of the cases in *D*_val_ have smaller Total Uncertainty. This corresponds to the 0.8 quantile of {*H*(*Y*|*X,D*) : (*X,Y*) ∈ *D*_val_}. We deemed 80% the most appropriate coverage to remain clinically useful. However, coverage could be further traded for performance with smaller cutoff values.

### Classification performance metrics and estimation

Classification performance is measured in terms of: Coverage, area under the reproducer-operator-curve (AUROC), accuracy (ACC), positive predictive value (PPV), negative predictive value (NPV), sensitivity and specificity. These metrics were estimated by 5-fold stratified cross-validation in the internal cohort. More concretely, for each fold: the corresponding training set is used to estimate the model and the selective classification cutoff; the corresponding test set is used to estimate internal cohort performance (table 3); and the entire external cohort is used to estimate external cohort performance (table 2). This leads to a total of 5 samples of performance. For each metric, we report the mean (*μ*) and two times the standard error (2*σ*). For reference, we also reported the prevalence of ACS in the test data, as this affects PPV and NPV.

#### Classification performance of ACS

The label for this task is either presence or absence of ACS. ACS is the acute manifestation of coronary heart disease and includes ST-elevation myocardial infarction (STEMI), non-ST elevation myocardial infarction (NSTEMI), and unstable angina (UA). Table 2 compares the ACS predictive performance of GBDT^10^, GBDT+SC, and the reported performance from alternative methods^1,2^. GBDT provides better predictive performance as noted by 24% improvement in sensitivity and 13% improvement in specificity. The rest of the metrics follow suit, with only PPV as the exception. The reason for the exception is that PPV can be arbitrarily high due to prevalence. In this case, even though (Takeda, 2022)^2^ discriminator is worse, their higher ACS prevalence masks this in the PPV. With respect to similar prevalence like (Al-Zaiti, 2020)^1^, our PPV is considerably better. Selective classification (SC) further improves performance (see first row in table 2) by filtering out uncertain cases (i.e. *H*(*Y*|*X,D*) > cutoff). For the filtered-in subpopulation of the external cohort, sensitivity and specificity improve by 4% and 10% points respectively, creating a considerable difference with respect to (Takeda, 2022) and (Al-Zaiti, 2020). Table 3 showcases slightly better performance for the internal cohort. This is expected as the model and cutoff are estimated from this cohort. Nevertheless, performance is similar with respect to the external cohort. This result suggests machine learning uncertainty estimates correlate with predictive performance, and that constraining predictions to a subset of patients, may reassure the model’s prediction.

#### Classification performance of NSTE-ACS

The label for this task is either presence or absence of ACS derived from NSTE-ACS. Like (Al-Zaiti, 2020)^1^, we consider NSTE-ACS as the presence of non-ST elevation MI or unstable angina. Table 4 compares the NSTE-ACS predictive performance of GBDT^10^ and the reported performance in (Al-Zaiti, 2020)^1^; (Takeda, 2022)^2^ did not report NSTE-ACS performance due to low prevalence (3.2%). For reference, we also included the prevalence of NSTE-ACS in the test samples, as this inflates/deflates certain metrics (e.g. PPV and NPV). GBDT improves both sensitivity and specificity by 14% and 7% respectively.

Like the ACS task, selective classification further improves performance by reducing coverage to 80%. For this subpopulation of the test set, average sensitivity and average specificity improve by 2% and 8 % points respectively. Results reinforce the notion that machine learned uncertainty estimates correlate with predictive performance, and that constraining predictions to a subset of patients may reassure us in the model’s prediction. This lead us to only suggest GBDT for this task if prevalence is close to 7%. Note this was not the case for the dataset used in (Al-Zaiti, 2020)^1^nor the dataset used in (Takeda, 2022)^2^.

### Ablations

#### What impact do input covariates have on performance?

The more covariates we consider, the more performance improves. We ablate the impact that different sources of data have in the classification (See table 5). Baseline corresponds to age, sex and ECG interpretations; Baseline + Symptoms correponds to all the baseline covariates and the symptoms covariates in table 1; Baseline + Symptoms + Medical History correponds to all baseline covariates, all symptoms covariates and all medical history covariates in table 1. As expected, performance increases the more covariates we consider. However, we observe a larger increase in sensitivity when we include Medical History.

#### Does total uncertainty correlate with performance?

**Figure 1.**
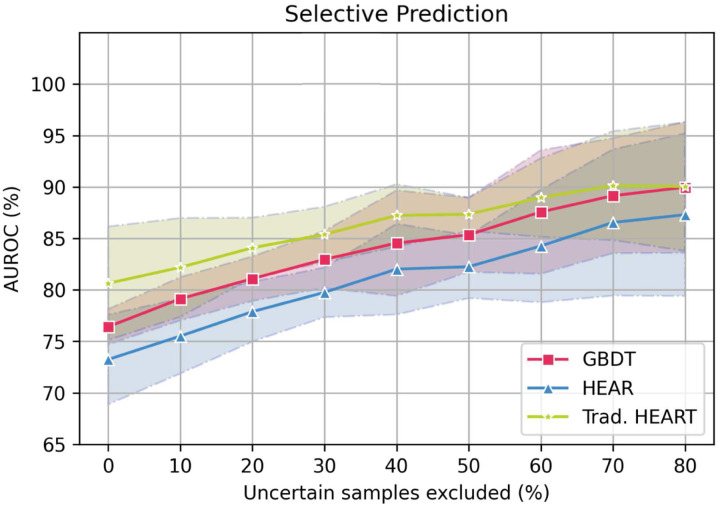
AUROC performance for three different methods (GBDT, HEAR^13^, HEART^12^) as we exclude more uncertain cases. Performance is computed with the non-excluded cases. Uncertainty is the predictive uncertainty from GBDT (Eq. 1). Highlighted is the mean and shaded is two standard deviations from 5-fold cross validation. For this experiment, GBDT uses less covariates than those in table 1 to match the HEAR^13^ covariates. Traditional HEART^12^ requires troponin, in addition to the HEAR covariates. A troponin measurement is generally unavailable in the prehospital setting.

Figure 1, red line with squares, suggest a positive correlation between the average performance of GBDT (y-axis) and the percentage of uncertain samples excluded (x-axis). As expected since excluding uncertain samples should mitigate errors.

#### Does total uncertainty correlate with performance of other ACS classifiers?

Figure 1 suggests the AUROC performance of unrelated methods (HEAR and HEART) correlates with the percentage of samples excluded. This assesses whether the excluded samples are deemed uncertain by other predictors. It is surprising that this is the case for both the HEAR and the HEART scores. This is important because we may use GBDT for patient selection and a different method for classification. For instance, the benefit of choosing HEAR and HEART as the classifier is that their prediction is explainable, a valuable feature for healthcare providers. Samples are excluded using the predictive uncertainty estimated from GBDT (Eq. 1). The larger the percentage of uncertain samples excluded, the higher performance we expect.

#### Does GBDT outperform other uncertainty quantification methods on ACS prediction?

We repeated the ACS classification experiment with two other popular approaches for uncertainty quantification (i.e. Deep Ensembles^16^ and MCDropout^17^). Results in table 6 suggest GBDT performs best. Posterior predictive entropy (i.e. *H*(*Y*|*X,D*) or total uncertainty) was used for selective classification across all methods. Note hyperparameter grid search was used for all methods.

#### Does GBDT outperform other prediction methods?

It depends (See table 7). If we are interested in rule-out performance (i.e. sensitivity and NPV) the answer is yes. If we are interested in rule-in performance (i.e. specificity and PPV), then XGBoost+SC is superior. Since rule-out performance is more desirable than the rule-in performance, and GBDT is designed for uncertainty quantification, we lean towards GBDT over XGBoost. For the experiments we repeated the ACS classification experiment with XGBoost and its corresponding predictive entropy (i.e. *H*(*Y*|*X*)) was used for selective classification. Note hyperparameter grid search was used for all methods.

## Discussion

In this study we measured the ACS and NSTE-ACS classification performance of GBDT and GBDT+SC. Results show that both methods achieve the best sensitivity and specificity reported for the prehospital setting. This is important because any method that furthers is a better candidate to aid the early rule-out or rule-in of ACS.

Compared to previously reported results^1,2^, GBDT^10^ is a better ML algorithm to rule-out ACS, with a 3% improvement in NPV and a 13% improvement in sensitivity. Selective classification (SC) further improves both rule-in and rule-out performance, with a 23% improvement in specificity, a 7% improvement in PPV, a 17% improvement in sensitivity and a 5% improvement in NPV. Nevertheless, selective classification introduces a tradeoff: on average, coverage (i.e. percentage of filtered-in test cases) reduces from 100% to 75% (See table 2). For the task of NSTE-ACS, the performance narrative is similar to the ACS case. GBDT provides the best rule-out performance with a 14% increase in sensitivity and a 5% increase in NPV. Selective classification further improves both rule-in and rule-out performance, with an increase of 16% sensitivity, 5% in NPV, 15% in specificity and a 6% increase in PPV. NSTE-ACS is important because it represents patients without ST-Elevation, a naturally ambiguous class of patients difficult to triage from ECG alone (See table 4). Methodologically, the main difference with respect to previous work is the ML model used for prediction. GBDT^10^ is designed for predictive uncertainty quantification, whereas previous methods^1,2^ are designed for predictive accuracy. This difference in design permits more elaborate decision making through the identification of the uncertainty source (See equation 3). Furthermore, we observe GBDT has better rule-out performance than the best previously found predictor^2^ (See table 7). In regards to input covariates, previous work^2^, we consider symptoms, an interpretation of the ECG and age in our prediction of ACS. However, we did not consider vital signs. We conjecture the addition of vital signs would improve performance like symptoms and history did in table 5; Unlike other works^1^, our methodology requires EMS personnel to interpret the ECG and determine the presence/absence of three conditions (See table 1). However, given how blackbox predictors are prone to random errors^18^ and overconfidence^19^, we argue EMS personnel should interpret the patient’s ECG, especially when rule-in and rule-out performance is insufficient. With respect to leveraging uncertainty in cardiovascular disease prediction outside the prehospital setting^4–6,20–23^, we also observed a positive correlation between selective classification and performance^4–6,23^. However, the deep learning methods^16,17^ employed among most these studies^4,5,20,21^ are outperformed by GBDT in this task (See table 6) and have more complex implementation. Additionally, we reemphasize deep learning models are generally unpredictable under imperceptible or irrelevant changes to the input signal^18,24^. Even though there have been studies that quantify decreases in performance due to changes in the input (e.g. Modifying the ECG SNR^23^, ECG distribution shift^4,5^), no guarantee exists these predictions will not be random or overconfident. Accordingly, as mentioned before, we encourage keeping the ECG interpreter in the loop^21^ until predictive performance is at desired levels^3^.

Whilst we showcase performance improvements, the work is still limited by various factors. First, a prospective study must be done to better ground the performance of any classifier trained retrospectively, especially one that may affect the course of treatment early in the care plan (e.g. GBDT+SC). Second, GBDT+SC prediction remains unexplainable, thus compromising clinical trust and downstream decision making^21^. There exist other explainable predictors that use a subset of our covariates to predict ACS^12,13^ but either the rule-in and rule-out performance is worse, or require Troponin, a measurement generally unavailable in the prehospital setting (See figure 1). In conclusion, we advocate GBDT with selective classification (i.e. GBDT+SC) to aid ACS screening in the prehospital setting. The improvement in predictive performance outweighs the loss in coverage for this task due to the sensible nature of the prediction. This advocation is stronger if the automatic classification is also intended to aid diagnosis, which requires better rule-in.

## Methods

### Machine Learning model development evaluation

Performance samples are obtained using 5-fold cross validation over the internal cohort to estimate a model and the external cohort to test it. That is, 5 different times, we splits the internal cohort into 20% for internal testing and 80% for training. We further split 10% of the internal training set for validation (*D*_*val*_), and leave the remaining for training (*D*). The validation set is used for hyper parameter search and the estimation of the selective classification cutoff. Hyperparameters are selected automatically using the grid_search function to mitigate selection biases (See table 8 for the grid space). The cutoff value is estimated as the 0.8 quantile of the total uncertainty estimates in the validation set. The GBDT method is implemented in the Catboost library, as class CatboostClassifier. During training and grid search, samples are weighted inversely proportional to the frequency of its corresponding class (see class_weights). The class weights are estimated dynamically from the data available for training in each cross-validation fold.

The following metrics are evaluated on the test set: coverage, area under the receiver operating curve (AUROC), accuracy (ACC), positive predictive value (PPV), negative predictive value (NPV), sensitivity and specificity. In the case of selective classification, these metrics are evaluated on a subset of the test set. This subset corresponds to the test cases that are smaller than the 0.8 quantile of the total uncertainty in the validation set. This is to guarantee around 80% of the test set is covered. The compromise in coverage was arbitrarily limited to 20%, mainly to preserve utility of automatic classification. Total uncertainty is defined in eq. (1) and is estimated by the average output entropy as in eq. (2). Where the average is across the output of all GBDT in the ensemble.

### Study population

The dataset used in this paper was obtained from the Optimizing Electrocardiographic Methods for the Early Identification of ST-Elevation Myocardial Infarction in Prehospital Cardiac Care study. The cohort study included patients transported by emergency medical services (EMS) with chest pain and/or anginal equivalent to Carolinas Medical Center (now Atrium) in Charlotte, NC (2013–2017). Per prehospital protocol, EMS providers obtained a standard 12-lead ECG on patients with suspected ACS. Raw digital ECG data were acquired and linked to hospital based clinical outcomes.

This study was approved by the institutional review board of the University of North Carolina at Chapel Hill, and all relevant ethical regulations on human experiments, including the declaration of Helsinki, have been followed. Data were collected through a healthcare registry, and all consecutive eligible patients were enrolled under a waiver of informed consent approved by the institutional review board of the University of North Carolina at Chapel Hill.

## Figures and Tables

**Table 1.** Statistics of covariates used as input to the Machine Learning model GBDT. Statistics are calculated separetely for the internal and external cohorts. For the ECG interpretations, type {0,1}^11^ indicates a binary vector. The position corresponds to the ECG lead used for the interpretation.

Characteristic	Type	Internal(n=1756)	External(n=1127)
Age	Numerical	61(±31)	60(±31)
Gender(male)	Binary	936(53%)	629(55%)
Medical History	Type	Internal(n=1756)	External(n=1127)
Hypercholesterolemia	Binary	693(39%)	485(43%)
Hypertension	Binary	943(53%)	803(71%)
Current Smoker	Binary	368(20%)	283(25%)
Diabetes	Binary	509(28%)	354(31%)
Prior MI	Binary	303(17%)	245(21%)
Angina	Binary	42(2%)	80(7%)
Prior CABG	Binary	166(9%)	180(15%)
Prior PCI	Binary	124(7%)	6(<1%)
CAD	Binary	349(19%)	271(24%)
Family history of CV disease	Binary	204(11%)	81(7%)
Symptoms	Type	Internal(n=1756)	External(n=1127)
Other	Binary	1753(99%)	1124(99%)
Chestpain	Binary	992(56%)	644(57%)
Syncope	Binary	103(5%)	69(6%)
Shortness of breath	Binary	417(23%)	282(25%)
Diaphoresis	Binary	114(6%)	89(7%)
Nausea and/or vomiting	Binary	164(9%)	113(10%)
Palpitations	Binary	226(12%)	164(14%)
Other symptoms	Binary	873(49%)	618(54%)
ECG Interpretation	Type	Internal(n=1756)	External(n=1127)
ST elevation	{0,1}^11^	329.0(18%)	170.0(15%)
ST depression	{0,1}^11^	500.0(28%)	217.0(19%)
T wave inversion	{0,1}^11^	252.0(14%)	180.0(15%)

**Table 2.** ACS classification performance on the external cohort. Reported is *μ* ±2*σ* where the samples come from 5-fold stratified cross-validation. For (Al-Zaiti)^1^ and (Takeda)^2^, the results presented are their reported results.

Method	Prevalence	Coverage	Sensitivity	Specificity	PPV	NPV	AUROC	Accuracy
GBDT+SC	16±2	75±5	94±2	96±4	81±10	99±0	95±2	95±3
GBDT	18±0	100±0	90±4	86±3	59±4	97±1	88±1	87±2
(Takeda, 2022)	48	100	86	73	74	86	82	79
(Al-Zaiti, 2020)	18	100	77±8	76±4	43±5	94±2	82±4	N/A

**Table 3.** ACS classification performance in the internal cohort. Reported is *μ* ±2*σ* where the samples come from 5-fold cross-validation.

Method	Prevalence	Coverage	Sensitivity	Specificity	PPV	NPV	AUROC	Accuracy
GBDT+SC	17±3	78±6	94±7	97±3	87±13	99±1	95±2	96±2
GBDT	20±0	100±0	88±7	89±6	68±12	97±2	89±4	89±5
(Takeda, 2022)	35	100	76	82	71	87	86	80
(Al-Zaiti, 2020)	15	100	N/A	N/A	N/A	N/A	N/A	N/A

**Table 4.** NSTE-ACS classification performance on the external cohort. Reported is *μ* ±2*σ* where the samples come from 5-fold stratified cross-validation.

Method	Prevalence	Coverage	Sensitivity	Specificity	PPV	NPV	AUROC	Accuracy
GBDT+SC	6±2	80±6	88±10	91±7	42±11	99±0	90±2	91±6
GBDT	7±0	100±0	86±10	83±4	28±3	99±1	85±3	83±3
(Al-Zaiti, 2020)	15	100	72±9	76±4	36±5	94±1	82±4	N/A
(Takeda, 2022)	3.2	N/A	N/A	N/A	N/A	N/A	N/A	N/A

**Table 5.** ACS classification performance for different input covariates: Baseline (i.e. ECG interpretations, Age and Sex); Baseline and Symptoms; Baseline, Symptoms and Medical History. Reported is *μ* ±2*σ* where the samples come from 5-fold cross-validation.

Metric (%)	Baseline	Baseline+Symptoms	Baseline+Symptoms+MedicalHistory
Sensitivity	63±5.1	67±3.7	89±4.4
Specificity	85±4.7	86±5.0	88±3.3
PPV	51±7.3	54±8.7	64±9.5
NPV	90±2.9	92±2.4	97±1.5
AUROC	74±4.2	77±2.1	88±1.4
Accuracy	81±4.8	83±3.9	88±2.0

**Table 6.** ACS classification performance on the external cohort. Reported is *μ* ±2*σ* where the samples come from 5-fold stratified cross-validation on the training set.

Method	Prevalence	Coverage	Sensitivity	Specificity	PPV	NPV	AUROC	Accuracy
GBDT	18±0	100±0	90±4	86±3	59±4	97±1	88±1	87±2
GBDT+SC	16±2	75±5	94±2	96±4	81±10	99±0	95±2	95±3
DeepEnsemble	18±0	100±0	87±6	84±6	55±8	97±1	85±2	84±4
DeepEnsemble+SC	19±2	76±7	92±4	90±7	70±15	98±1	91±4	91±6
MCDropout	18±0	100±0	75±26	90±11	65±16	95±5	83±9	87±6
MCDropout+SC	14±7	79±5	82±24	96±8	79±21	98±2	89±10	94±6

**Table 7.** ACS classification performance on the external cohort. Reported is *μ* ±2*σ* where the samples come from 5-fold stratified cross-validation on the training set. XGBoost is the predictor with the best reported performance in previous work^2^

Method	Prevalence	Coverage	Sensitivity	Specificity	PPV	NPV	AUROC	Accuracy
GBDT	18±0	100±0	90±4	86±3	59±4	97±1	88±1	87±2
GBDT+SC	16±2	75±5	94±2	96±4	81±10	99±0	95±2	95±3
XGBoost	18±0	100±0	63±12	97±3	83±13	92±2	80±4	91±1
XGBoost+SC	11±2	80±6	70±10	99±0	93±2	97±1	85±5	96±1

**Table 8.** Hyperparameters for both ACS and NSTE-ACS tasks

Catboost Hyperparameter	Space
learning_rate	[0.01, 0.1, 1.0]
depth	[1, 3, 6, 10]
subsample	[0.25, 0.5, 0.75]
iterations	[1000]
border_count	[128]
random_strength	[0]
bootstrap_type	[‘Bernoulli’]
posterior_sampling	[True]
random_seed	[1419528]
nensemble	[10]
class_weights	[.,.]

## Data Availability

The datasets generated during and/or analysed during the current study are not publicly available due to institutional data use agreements but are available from Jessica K. Zègre-Hemsey PI (jzhemsey@email.unc.edu) on reasonable request.

## References

[1] Al-Zaiti S. Machine learning-based prediction of acute coronary syndrome using only the pre-hospital 12-lead electrocardiogram. Nat. Commun. 11, 3966, DOI: 10.1038/s41467-020-17804-2 (2020). 32769990 PMC7414145

[2] Takeda M. Prehospital diagnostic algorithm for acute coronary syndrome using machine learning: a prospective observational study. Sci. Reports 12, 14593, DOI: 10.1038/s41598-022-18650-6 (2022). PMC9418242 36028534

[3] Cooper J. G. Performance of a prehospital heart score in patients with possible myocardial infarction: a prospective evaluation. Emerg. Medicine J. 40, 474–481, DOI: 10.1136/emermed-2022-213003 (2023). 37268413

[4] Barandas M. Evaluation of uncertainty quantification methods in multi-label classification: A case study with automatic diagnosis of electrocardiogram. Inf. Fusion 101, 101978, DOI: 10.1016/j.inffus.2023.101978 (2024).

[5] Vranken J. F. Uncertainty estimation for deep learning-based automated analysis of 12-lead electrocardiograms. Eur. Hear. Journal-Digital Heal. 2, 401–415, DOI: 10.1093/ehjdh/ztab045 (2021). PMC9707930 36713602

[6] Upadhyay U. Hypuc: Hyperfine uncertainty calibration with gradient-boosted corrections for reliable regression on imbalanced electrocardiograms. Transactions on Mach. Learn. Res. (2023).

[7] Cordella L., De Stefano C., Tortorella F. & Vento M. A method for improving classification reliability of multilayer perceptrons. IEEE Transactions on Neural Networks 6, 1140–1147, DOI: 10.1109/72.410358 (1995). 18263404

[8] El-Yaniv R. On the foundations of noise-free selective classification. J. Mach. Learn. Res. 11 (2010).

[9] Filos A. A systematic comparison of bayesian deep learning robustness in diabetic retinopathy tasks. arXiv:1912.10481 [cs, eess, stat] (2019). ArXiv: 1912.10481.

[10] Malinin A., Prokhorenkova L. & Ustimenko A. Uncertainty in gradient boosting via ensembles. arXiv:2006.10562 [cs, stat] (2021). ArXiv: 2006.10562.

[11] Zègre-Hemsey J. K. Prehospital ecg with st-depression and t-wave inversion are associated with new onset heart failure in individuals transported by ambulance for suspected acute coronary syndrome. J. Electrocardiol. 69, 23–28 (2021). 10.1016/j.jelectrocard.2021.08.005 PMC8665024 34456036

[12] Backus B. A prospective validation of the heart score for chest pain patients at the emergency department. Int. J. Cardiol. 168, 2153–2158, DOI: 10.1016/j.ijcard.2013.01.255 (2013). 23465250

[13] Stopyra J. P. Prehospital modified heart score predictive of 30-day adverse cardiac events. Prehospital Disaster Medicine 33, 58–62, DOI: 10.1017/S1049023X17007154 (2018). 29316995

[14] Le Morvan M., Josse J., Scornet E. & Varoquaux G. What’s a good imputation to predict with missing values? In Ranzato M., Beygelzimer A., Dauphin Y., Liang P. S. & Vaughan J. W. (eds.) Advances in Neural Information Processing Systems, vol. 34, 11530–11540 (Curran Associates, Inc., 2021).

[15] Malinin A. Uncertainty estimation in deep learning with application to spoken language assessment. Ph.D. thesis, University of Cambridge (2019).

[16] Fort S., Hu H. & Lakshminarayanan B. Deep ensembles: A loss landscape perspective. arXiv preprint arXiv:1912.02757 (2019).

[17] Gal Y. & Ghahramani Z. Dropout as a bayesian approximation: Representing model uncertainty in deep learning. In international conference on machine learning, 1050–1059 (PMLR, 2016).

[18] Sato M., Suzuki J., Shindo H. & Matsumoto Y. Interpretable adversarial perturbation in input embedding space for text. arXiv preprint arXiv:1805.02917 (2018).

[19] Guo C., Pleiss G., Sun Y. & Weinberger K. Q. On calibration of modern neural networks. In International conference on machine learning, 1321–1330 (PMLR, 2017).

[20] Aseeri A. O. Uncertainty-aware deep learning-based cardiac arrhythmias classification model of electrocardiogram signals. Computers 10, 82, DOI: 10.3390/computers10060082 (2021).

[21] Elul Y., Rosenberg A. A., Schuster A., Bronstein A. M. & Yaniv Y. Meeting the unmet needs of clinicians from ai systems showcased for cardiology with deep-learning–based ecg analysis. Proc. Natl. Acad. Sci. 118, e2020620118, DOI: 10.1073/pnas.2020620118 (2021). 34099565 PMC8214673

[22] Park J., Lee K., Park N., You S. C. & Ko J. Self-attention lstm-fcn model for arrhythmia classification and uncertainty assessment. Artif. Intell. Medicine 142, 102570, DOI: 10.1016/j.artmed.2023.102570 (2023). 37316094

[23] Jahmunah V., Ng E., Tan R.-S., Oh S. L. & Acharya U. R. Uncertainty quantification in densenet model using myocardial infarction ecg signals. Comput. Methods Programs Biomed. 229, 107308, DOI: 10.1016/j.cmpb.2022.107308 (2023). 36535127

[24] Hein M., Andriushchenko M. & Bitterwolf J. Why relu networks yield high-confidence predictions far away from the training data and how to mitigate the problem. In Proceedings of the IEEE/CVF Conference on Computer Vision and Pattern Recognition, 41–50, DOI: 10.1109/CVPR.2019.00013 (2019).

